# Heavy metal tolerance in Scopelophila cataractae: Transcriptomic and epigenetic datasets

**DOI:** 10.1016/j.dib.2022.108710

**Published:** 2022-10-28

**Authors:** M. Teresa Boquete, Marc W. Schmid, Niels C.A.M. Wagemaker, Sarah B. Carey, Stuart F. McDaniel, Christina L. Richards, Conchita Alonso

**Affiliations:** a Area of Ecology, Department of Functional Biology, Universidade de Santiago de Compostela, Lope Gómez de Marzoa s/n, 15705, Spain; b Department of Evolutionary Ecology, Estación Biológica de Doñana, CSIC, Americo Vespucio 26, 41092 Sevilla, Spain; c Department of Integrative Biology, University of South Florida, 4202 East Fowler Ave, 33620, Tampa, FL, USA; d MWSchmid GmbH, Hauptstrasse 34, CH-8750 Glarus, Switzerland; e Faculty of Science, Raboud University, Heyendaalseweg 135, 6525, AJ Nijmegen, the Netherlands; f Department of Biology, University of Florida, 876 Newell Dr, 32611, Gainesville, FL, USA; g Plant Evolutionary Ecology group, University of Tübingen, Auf der Morgenstelle 5, D-72076 Tübingen, Germany

**Keywords:** Abiotic stress, Bryophytes, Copper moss, DNA methylation, Gene expression, Intraspecific phenotypic variation

## Abstract

Studying how different plant groups deal with heavy metal exposure is crucial to improve our understanding of the diversity of molecular mechanisms involved in plant stress response. Here, we used RNA sequencing (RNA-seq) and epigenotyping by sequencing (epiGBS) to assess gene expression and DNA methylation changes respectively in plants from four populations of the metallophyte moss Scopelophila cataractae treated with Cd or Cu in the laboratory. We built RNA-seq and epiGBS sequencing libraries from control and treated samples from each population and sequenced them using Illumina HiSeq 3000 (PE-150 bp) and Illumina HiSeq X-Ten System (PE-150 bp) respectively. For the RNA-seq data, we performed a read quality filter, mapped the reads to the de novo transcriptome created with Trinity, and estimated transcript abundance for each sample. For the epiGBS data, we used a custom pipeline (https://doi.org/10.5281/zenodo.7040291) to map the reads to a de novo reference genome and performed strand-specific nucleotide (single nucleotide polymorphisms, SNPs) and methylation (single cytosine methylation polymorphisms, SMPs) variant calling. We filtered out SNPs and SMPs with low coverage within (positions with <10 sequencing reads per sample) and across samples (positions with poor representation on the full set of samples). Finally, we performed pairwise comparisons between control and treated samples from each population and identified differentially expressed genes and differentially methylated cytosines associated to heavy metal exposure. We payed particular attention to the different responses of the more and the less tolerant populations of S. cataractae. These datasets could contribute to future comparative studies of abiotic stress response across plant groups.

**Specifications Table**

**Table:** 

Subject	Plant Science: General
Specific subject area	Transcriptomic and epigenetic changes during heavy metal exposure; abiotic stress response in plants
Type of data	Raw fastq files Processed fasta files Tables
How the data were acquired	Samples: extraction of total RNA and genomic DNA from control and treated plants. Data: RNA sequencing (RNA-seq) with Illumina HiSeq 3000 (PE-150 bp) and epigenotyping by sequencing (epiGBS) with Illumina HiSeq X-Ten System (PE-150 bp). Software RNA-seq: Trimmomatic v 0.36, Trinity v 2.8.4, CD-HIT v 4.6.6, Bowtie2 v 2.2.9, BUSCO v 3.1.0, RSEM, OmicsBox Software epiGBS: epiGBS custom pipeline (https://doi.org/10.5281/zenodo.7040291), RepeatMasker v 4.0, DIAMOND v 0.8.22.
Data format	Raw data: Illumina FASTQ files for epiGBS (paired end reads), and for RNAseq (individual samples’ Illumina paired end reads after quality trimming), and barcode file (barcodes.tsv) for epiGBS data submitted to the Sequence Read Archive (SRA) of NCBI under project number PRJNA790924. Processed data: reference contigs (de_novo_contigs.fasta.gz), annotation of the reference contigs (mergedAnnot.csv.gz), SNPs (snps.vcf.gz), methylation data (methylation.txt.gz), de novo transcriptome assembly (de_novo_transcriptome.fasta.gz), and de novo transcriptome assembly annotation (de_novo_transcriptome_annotation_blast&IPS.txt) Analysed data: supplementary tables (SupplementaryMaterials_Boquete_et_al_DiB.tar.gz)
Description of data collection	Gametophore tissue was collected from control, Cd-treated and Cu-treated plants of four populations of S. cataractae (3 biological replicates per treatment and population) and subjected to genomic DNA extraction. A subset of control and Cu-treated plants from two of the populations were subjected to total RNA extraction. RNA-seq and epiGBS libraries were sequenced on Illumina HiSeq 3000 and Illumina HiSeq X-Ten System respectively to obtain 150 bp paired-end reads.
Data source location	• Institution: Estación Biológica de Doñana (EBD-CSIC) • City/Town/Region: Sevilla, Andalucía • Country: Spain • Latitude and longitude (and GPS coordinates, if possible) for collected samples/data: 17N 572397E 3951654N
Data accessibility	Raw sequencing data and barcodes file can be accessed from NCBI SRA (BioProject ID: PRJNA790924) https://www.ncbi.nlm.nih.gov/bioproject/PRJNA790924 epiGBS custom scripts: can be found on zenodo (https://doi.org/10.5281/zenodo.7040291) [1] Processed data: reference contigs, annotation of the reference contigs, SNPs, methylation data, de novo transcriptome assembly, and de novo transcriptome assembly annotation are available on zenodo (https://doi.org/10.5281/zenodo.6695117) [2] Analysed data: supplementary tables are available on zenodo (https://doi.org/10.5281/zenodo.6695117) [2]
Related research article	Boquete, M.T., Schmid, M.W., Wagemaker, N.C.A.M., Carey, S.B., McDaniel, S.F., Richards, C.L., Alonso, C. Molecular basis of intraspecific differentiation for heavy metal tolerance in the copper moss Scopelophila cataractae. Environ. Exp. Bot. 201 (2022) 104970. https://doi.org/10.1016/j.envexpbot.2022.104970

## Value of the Data

• The transcriptomic data provide a detailed comparative view of gene expression changes in plants of the metallophyte moss Scopelophila cataractae with different Cu tolerance levels. The molecular mechanisms involved in intraspecific differentiation for Cu tolerance can thus be inferred.
• The epigenetic data provides a unique source of information about changes in DNA methylation in response to abiotic stress in bryophytes.
• Both datasets could be used by plant scientists to perform comparative studies of the evolution of plant abiotic stress responses.
• These data could also contribute to the identification of target genes for the development of biotechnological tools in the field of phytoremediation.

## 1 Data Description

An overview of the raw epigenetic and transcriptomic data available on the Sequence Read Archive (SRA) can be found in Table 1. The epigenetic dataset was obtained by extracting genomic DNA from control, Cd- and Cu-treated plants from four populations of the metallophyte moss Scopelophila cataractae that were collected in the field where they were exposed to different levels of heavy metals. The transcriptomic dataset was obtained from total RNA extracted from a subset of the previous sample set that included control and Cu-treated plants from two of the four populations (one tolerant and one sensitive population of S. cataractae). The plants were treated in the laboratory for 30 days, then DNA and RNA were extracted, and the sequencing libraries were prepared as described in Section 2.

**Table 1:** Sample names and additional information of the raw epigenetic (epiGBS sequencing reads) and transcriptomic (RNA-seq) datasets obtained from gametophore samples of the moss Scopelophila cataractae subjected to control (plants watered with deionized - DI - water), Cd, and/or Cu treatments (plants watered with DI water containing either 0.1 mM Cd or 1 mM Cu) available on the Sequence Read Archive (SRA). Multiplexed raw fastq files (R1 and R2 corresponding to paired-end sequencing) correspond to the epigenetic data; individual samples’ R1 and R2 file correspond to the transcriptomic data.

SRA sample name	Population	Treatment	SRA library ID	File type	Filename (R1)	Filename (R2)
Scopelophila_cataractae_epiGBS	Sc1, Sc2, Sc3, Sc4	Control, Cd-treated, Cu-treated	Sc_epiGBS	fastq	Sc_EpiGBS_R1.fq.gz	Sc_EpiGBS_R2.fq.gz
10	Sc3	Control	Sc3_C1_RNAseq	fastq	10_R1_paired.fq.gz	10_R2_paired.fq.gz
21	Sc3	Control	Sc3_C2_RNAseq	fastq	21_R1_paired.fq.gz	21_R2_paired.fq.gz
29	Sc3	Control	Sc3_C3_RNAseq	fastq	29_R1_paired.fq.gz	29_R2_paired.fq.gz
16	Sc4	Control	Sc4_C1_RNAseq	fastq	16_R1_paired.fq.gz	16_R2_paired.fq.gz
30	Sc4	Control	Sc4_C2_RNAseq	fastq	30_R1_paired.fq.gz	30_R2_paired.fq.gz
38	Sc4	Control	Sc4_C3_RNAseq	fastq	38_R1_paired.fq.gz	38_R2_paired.fq.gz
22	Sc3	Cu-treated	Sc3_Cu1_RNAseq	fastq	22_R1_paired.fq.gz	22_R2_paired.fq.gz
33	Sc3	Cu-treated	Sc3_Cu2_RNAseq	fastq	33_R1_paired.fq.gz	33_R2_paired.fq.gz
34	Sc3	Cu-treated	Sc3_Cu3_RNAseq	fastq	34_R1_paired.fq.gz	34_R2_paired.fq.gz
9	Sc4	Cu-treated	Sc4_Cu1_RNAseq	fastq	9_R1_paired.fq.gz	9_R2_paired.fq.gz
23	Sc4	Cu-treated	Sc4_Cu2_RNAseq	fastq	23_R1_paired.fq.gz	23_R2_paired.fq.gz
28	Sc4	Cu-treated	Sc4_Cu3_RNAseq	fastq	28_R1_paired.fq.gz	28_R2_paired.fq.gz

**Fig. 1:**
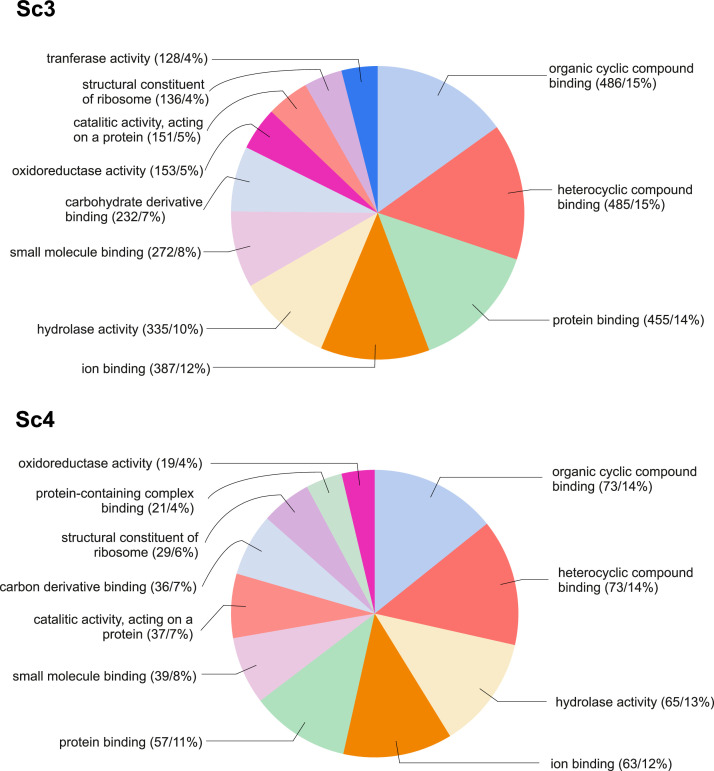
Pie chart of gene ontology (GO) terms related to all molecular functions represented in the list of differentially expressed transcripts (DETs) in response to Cu in Sc3 and Sc4 respectively. The number of DETs within each GO and the percentage from the total number of DETs are presented within brackets.

In total, 26,638 transcripts of the de novo transcriptome assembly were successfully annotated. The GO terms related to all molecular functions represented in the list of differentially expressed transcripts (DETs) in response to Cu in two of the studied populations (Sc3 and Sc4) is shown in Fig. 1.

**Fig. 2:**
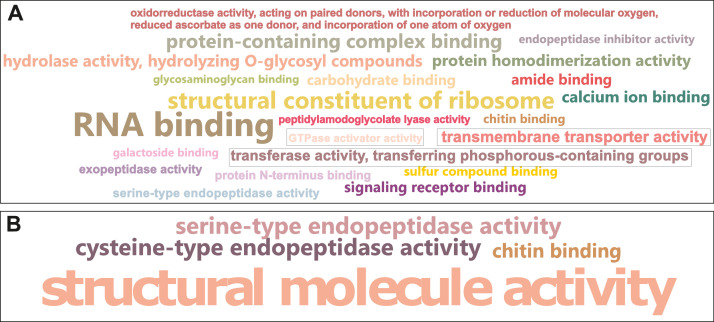
Word cloud representation of gene ontology (GO) terms related to all molecular functions significantly over-represented in the list of differentially expressed transcripts (DETs) in response to Cu in Sc3 (A; n=22 GOs) and Sc4 (B; n=4 GOs). The size of the words is proportional to the frequency of the GO term in the DETs list. GOs inside grey rectangles show under-represented terms in the DETs’ list.

Finally, Fig. 2 shows all the significantly over-represented GO terms in response to Cu exposure between the annotated list of differentially expressed transcripts (test set) and the full list of annotated transcripts (reference set) for each population of the S. cataractae (Sc3 and Sc4).

## 2 Experimental Design, Materials and Methods

### 2.1 Sample collection and laboratory treatments

We used plants from four populations of Scopelophila cataractae collected in the field where they were naturally growing under different levels of heavy metal contamination. These plants were clonally propagated in a growth chamber in the Department of Integrative Biology at the University of South Florida (Tampa, FL, USA) and treated with water (controls), water enriched with 0.1 mM Cd (Cd-treated) or water enriched with 1 mM Cu (Cu-treated) - 4-5 replicates per population and treatment - as described in [3]. After one month, we harvested all plants from the different treatments and split each sample into several aliquots: one to perform growth measurements [3], one to extract total RNA, and one to extract genomic DNA. The latter two aliquots were flash frozen in liquid nitrogen and stored at -80°C prior to extractions.

### 2.2 Genomic DNA extraction, epiGBS library preparation, sequencing, and filtering

We isolated genomic DNA (gDNA) from 36 samples (three biological replicates from each of control, Cd-treated, and Cu-treated plants) using the cetyltrimethylammonium bromide (CTAB) DNA extraction protocol for recalcitrant plant tissues (https://www.protocols.io/view/high-quality-dna-extraction-protocol-from-recalcit-i8jchun). Then, we checked the quality of the gDNA with the NanoDrop (Nanodrop™ 8000 Spectrophotometer; Thermo Scientific), and quantified its concentration using the Qubit 3.0 Fluorometric dsDNA BR assay kit (Q32851; Life Technologies).

We prepared the epiGBS libraries following [4] with 400 ng of gDNA per sample as starting material. First, we digested the gDNA with the restriction enzyme PstI; second, we ligated methylated, non-phosphorylated barcoded adapters to both ends of the digested fragments; third, we concentrated the library using the NucleoSpin™ Gel and PCR Clean-up Kit (12303368, Macherey-Nagel™) and performed a fragment size selection with 0.8x SPRI beads (A63880, Agencourt AMPure XP Beckman coulter); fourth, we performed nick translation, bisulfite conversion of the DNA using the EZ Lightning methylation kit (Zymo Research), and amplified the library with the KAPA HIFI Uracil+ Hotstart Ready Mix (Roche). Finally, we sequenced the libraries at Novogene (HK) Company Limited in Hong Kong on the Illumina HiSeq X-Ten System (PE-150 bp).

Using a custom pipeline, developed by [4] and modified as in https://doi.org/10.5281/zenodo.7040291, we demultiplexed the sequencing reads, quality trimmed them, and used these pooled reads to create a de novo reference genome assembly. Then, we individually mapped the reads from each sample to the assembly and performed strand-specific nucleotide (single nucleotide polymorphisms, SNPs) and methylation (single methylation polymorphisms, SMPs) variant calling. We recovered 85.8 million reads after demultiplexing with an average number of reads per sample of 2.5 million (ranging between 0.6-4.5 million).

The unfiltered datasets resulting from the epiGBS pipeline consisted of 279,103 SNPs and 22.3 million SMPs across all samples. We filtered these datasets as follows: (1) we removed SNPs and SMPs without a minimum coverage of 3 and a maximum coverage equal to the 99th percentile of the read coverage distribution, in at least one replicate sample per group. In total 58,773 SNPs and 290,547 SMPs were kept; (2) we identified low quality samples, i.e. samples lacking more than 20% of the SNPs or more than 25% of the SMPs; and (3) we removed these samples from the experimental design, used the original (unfiltered) SNP and SMP dataset and removed SNPs and SMPs without a minimum coverage of 10 and a maximum coverage equal to the 99th percentile of the read coverage distribution in at least one replicate sample per group (i.e. population x treatment combination). The last filtering step resulted in a final working dataset of 52,513 SNPs and 239,728 SMPs across 30 samples comprising 2-3 replicates per group.

### 2.3 Total RNA extraction, library preparation, sequencing, and filtering

We isolated total RNA from a subset of 12 samples, including three biological replicates from each of control and Cu-treated plants from two populations that had been exposed to different levels of heavy metals in the field and showed contrasting responses to Cu exposure in the laboratory, with the RNeasy Plant Mini Kit (74904, Qiagen) following the manufacturer instructions with small modifications First, we added 450 µl of disruption buffer (RTL) containing 1% BME to each frozen sample, vortexed them vigorously, and ground them in a TissueLyser. Then, we followed the manufacturer's protocol including the on-column DNase I digestion to remove DNA from all samples (Rnase-free DNase set, 79254, Qiagen). Then, we measured the concentration of total RNA in each sample using the Qubit RNA BR assay kit (Q10210, Invitrogen) and assessed the quality with the RNA integrity number (RIN) from the 2200 Tape Station (Agilent).

We prepared the cDNA libraries with the NEBNext^Ⓡ^ Ultra™ II Directional RNA Library Prep with Sample Purification Beads (E7765S, NEB) following the manufacturer's protocol with 1 µg of total RNA per sample as input material. Briefly, we isolated messenger RNA (mRNA) from total RNA using the NEBNext^Ⓡ^ Poly(A) mRNA Magnetic Isolation Module kit (E7490S, NEB). We indexed each library with a combination of two of the NEBNext^Ⓡ^ Multiplex Oligos for Illumina^Ⓡ^ (Dual Index Primers Set 1) (E7600, NEB), quantified the libraries using the Qubit dsDNA assay kit, pooled all libraries using equimolar concentrations, and checked their quality with the 2200 Tape Station (Agilent). Finally, we sequenced the libraries at the University of Florida Interdisciplinary Center for Biotechnology Research on the Illumina HiSeq 3000 (PE-150 bp).

We received individual raw sequencing reads files for each sample and quality trimmed them by removing adaptors and low-quality bases with Trimmomatic v 0.36 [5] with a 4-base sliding window and quality threshold of 25. The reads from each individual sample were pooled and used for de novo transcriptome reconstruction with Trinity v 2.8.4 [6], following the protocol by [7]. Then, we clustered highly redundant transcripts, i.e. transcripts with > 95% sequence similarity, using CD-HIT v 4.6.6 [8], and selected the longest isoform per gene using Trinity's custom script (get_longest_isoform_seq_per_trinity_gene.pl).

### 2.4 De novo transcriptome quality assessment and annotation

We assessed the quality of the de novo transcriptome assembly by mapping the trimmed reads back to the assembly with Bowtie2 v 2.2.9 [9] and evaluated its completeness by searching for orthologues with BUSCO v 3.1.0 [10] using the viridiplantae-odb10 as a reference database (E-value cutoff for the blast alignments: 1e^−06^).

We used the pipeline available within the bioinformatics platform OmicsBox [11,12] to annotate the de novo transcriptome.

### 2.5 Data analysis

All analyses were performed in R v.3.5.1 [13] running under R Studio v.1.2.5019 [14].

First, we tested for genetic differentiation among populations of S. cataractae by performing an analysis of molecular variance (AMOVA) with the function poppr.amova in the poppr R package [15] using the SNP data. We assessed the significance of the model using a randomization test with 9999 permutations on the output of the AMOVA (function randtest from the ade4 package [16]).

Second, we tested the effect of the heavy metal treatments on DNA methylation by performing a distance-based redundancy analysis (dbRDA [17]) on the matrix of pairwise epigenetic distances created from the SMP dataset following the formula presented by [18]. We run two separate dbRDA models, one testing the effect of Cu (epigenetic distance ∼ Population * Cu treatment) and one testing the effect of Cd (epigenetic distance ∼ Population * Cd treatment).

Third, we identified differentially methylated cytosine positions (DMPs) as cytosines with significant differences in their mean methylation level between control and Cu-treated, and control and Cd-treated plants using the R package DSS [19]. First, we modelled the methylation frequency at each cytosine position within each group using a beta-binomial distribution with arcsine link function and the formula “∼ 0 + group” (function DMLfit.multifactor). Then, we performed Wald tests to detect differential methylation between groups at each position using the function DMLtest.multifactor which reports adjusted p-values by the Benjamini-Hochberg method (i.e. FDR). We considered cytosines to be differentially methylated (i.e. DMPs) when FDR ≤ 0.05 and methylation change was ≥ 10%.

Fourth, we identified differentially expressed transcripts (DETs) between control and Cu-treated plants for each population as follows: (1) we estimated transcript abundance within each individual sample using RSEM [7] wrapped by scripts included in Trinity (align_and_estimate_abundance.pl); (2) we used edgeR v3.24.3 [20] to filter low count transcripts by removing those with less than 2 cpm (counts per million; i.e. less than 10 counts per transcript) in at least 3 samples; and (3) we fitted the model using the function glmQLFit (option robust = TRUE) and tested for DETs using the function glmTreat. Transcripts were considered differentially expressed when FDR < 0.001 and expression change was ≥ 4-fold (log2FC ≥ 2).

Finally, we performed a Fisher's exact test with the FatiGO package [21] on the list of DETs from each population to find differences in the fraction of transcripts annotated with a specific Gene Ontology (GO) term between the DETs list and the full list of annotated transcripts (reference set). We applied an FDR cutoff of 0.01, and used the “Reduce to most specific” option within OmicsBox to remove the more general, less informative, GO terms.

## Ethics Statements

This research did not involve human or animal subjects nor data collected from social media platforms.

## CRediT authorship contribution statement

**M. Teresa Boquete:** Conceptualization, Data curation, Formal analysis, Funding acquisition, Investigation, Methodology, Writing – original draft, Writing – review & editing. **Marc W. Schmid:** Data curation, Formal analysis, Writing – review & editing. **Niels C.A.M. Wagemaker:** Methodology. **Sarah B. Carey:** Methodology, Writing – review & editing. **Stuart F. McDaniel:** Methodology, Writing – review & editing. **Christina L. Richards:** Conceptualization, Supervision, Investigation, Methodology, Writing – review & editing. **Conchita Alonso:** Conceptualization, Supervision, Investigation, Methodology, Writing – review & editing.

## Declaration of Competing Interest

The authors declare that they have no known competing financial interests or personal relationships that could have appeared to influence the work reported in this paper.

The authors declare the following financial interests/personal relationships which may be considered as potential competing interests:

## Data Availability

Raw sequencing data and barcodes file for epiGBS (Original data) (NCBI SRA). Processed and analyzed data (Original data) (Zenodo). epiGBS custom scripts (Original data) (Zenodo).

## Appendix Supplementary materials

Image, application 1
