# Supplementary material for: Heavy metal tolerance in Scopelophila cataractae: Transcriptomic and epigenetic datasets
Source: Data Brief. 2022 Oct 28;45:108710. doi: 10.1016/j.dib.2022.108710 (PMC9679722; doi:10.1016/j.dib.2022.108710)
Supplement: Supplementary file 1 [file mmc1.zip › SupplementaryData_Boquete_et_al.tar.gz!/SupplementaryData_Boquete_et_al/SupplementaryMaterials.docx]

Authors: M. Teresa Boquete, Marc W. Schmid, Niels C.A.M. Wagemaker, Sarah B. Carey, Stuart F. McDaniel, Christina L. Richards, Conchita Alonso

Article title: Molecular basis of intraspecific differentiation for heavy metal tolerance in the copper moss *Scopelophila cataractae*.

Journal name: Environmental and Experimental Botany

**Supplementary Methods**

*1. DNA extraction protocol*

We followed the cetyltrimethylammonium bromide (CTAB) DNA extraction protocol for recalcitrant plant tissues (<https://www.protocols.io/view/high-quality-dna-extraction-protocol-from-recalcit-i8jchun>) with small modifications. First, we ground frozen samples to a fine powder for 3-4 minutes (TissueLyser, Qiagen) and added 1 ml of STE buffer (sucrose, Tris-HCl pH 8 (1M), EDTA (0.5M), and molecular water) to each sample. We vortexed the mixture thoroughly, centrifuged it at 5,000 rpm for 10 min, and discarded the supernatant. Then, we added 500 µL of pre-warmed CTAB buffer (Tris-HCL pH8 -1M; final conc. 100 mM-, NaCl -5M; final conc. 1.5M-, EDTA -0.5M; final conc. 25 mM-, CTAB -final conc. 2-3%-, PVP -final conc. 2%-, BME -final conc. 0.2%-, and molecular H_2_O), and 100 µL of NaCl (5M):BSA (4%) solution (5:1) to each tube. We vortexed the mixture and incubated it for 1h at 65ºC. We added 450 µL of chloroform-isoamyl alcohol (24:1), inverted the samples by hand for 5 min, and centrifuged them at maximum speed for 5 min. We recovered ~400 µL of the top aqueous phase to a new tube and carried out a second chloroform step. Again, we recovered ~400 µL of the top aqueous phase to a 1.5-mL tube, added 200 µL of NaCl (5M) and 900 µL of 95% ethanol, and incubated it for 20 min at room temperature to precipitate the DNA. We centrifuged the samples for 10 minutes at maximum speed and discarded the supernatant. Finally, we washed the gDNA with 500 µL of freshly prepared 70% ethanol, centrifuged it for 5 min at maximum speed, discarded the supernatant, dried the samples, and re-suspended the gDNA in 50 µL of molecular H_2_O.

*2. RNA extraction and RNAseq library preparation*

Total RNA was isolated with the RNeasy Plant Mini Kit (74904, Qiagen) following the manufacturer instructions with small modifications (detailed protocol available in the Supplementary Methods). First, we added 450 µl of disruption buffer (RTL) containing 1% BME to each frozen sample, vortexed them vigorously, and ground them in a TissueLyser. Then, we followed the manufacturer’s protocol including the on-column DNase I digestion to remove DNA from all samples (Rnase-free DNase set, 79254, Qiagen). We measured the concentration of total RNA in each sample using the Qubit RNA BR assay kit (Q10210, Invitrogen) and assessed the quality with the RNA integrity number (RIN) from the 2200 Tape Station (Agilent).

The cDNA libraries were prepared with the NEBNext® Ultra™ II Directional RNA Library Prep with Sample Purification Beads (E7765S, NEB) following the manufacturer’s protocol with 1 µg of total RNA as input material. Briefly, we isolated messenger RNA (mRNA) from total RNA using the NEBNext® Poly(A) mRNA Magnetic Isolation Module kit (E7490S, NEB). We indexed each library with a combination of two of the NEBNext® Multiplex Oligos for Illumina® (Dual Index Primers Set 1) (E7600, NEB), quantified the libraries using the Qubit dsDNA assay kit, pooled all libraries using equimolar concentrations, and checked their quality with the 2200 Tape Station (Agilent). We performed paired-end read sequencing (PE-150) at the University of Florida Interdisciplinary Center for Biotechnology Research on the Illumina HiSeq 3000.

**Supplementary Tables**

**Table S1**: Total number of sequencing reads (in million) per sample, group and population of the copper moss *Scopelophila cataractae* in the epiGBS dataset after demultiplexing. Bold sample IDs highlight samples discarded during the second step of the filtering (see section 2.5.1 of the manuscript). C: control samples; Cd: cadmium-treated samples; Cu: copper-treated samples.

**Table S2**: Table S2: Total number of sequencing reads (in million) per sample, population, and group of the copper moss *Scopelophila cataractae* in the RNA-seq dataset before (raw reads) and after (trimmed reads) quality trimming. C: control samples; Cu: copper-treated samples.

**Table S3**: Summary of the *de novo* transcriptome assembly quality (read mapping rate) and completeness (BUSCO single-copy ortholog content) assessment, as well as the gene ontology (GO) annotation. **Excel File**.

**Table S4**: Results of the distance-based redundancy analysis (dbRDA) carried out to test the effect of population, treatment, and their interaction on genome wide DNA methylation for each of the common garden experiments for each DNA sequence context separately (CG, CHG, CHH), and across all contexts together (all). The results of the permutation test (n = 9999) for the significance of each of the predictors are also shown (Predictors). This analysis was run on two different response variables: the pairwise distance matrix calculated with the methylation level only ($d_{st}^{m}$), and the pairwise distance matrix calculated with the methylation level and variance ($d_{st}^{m\&v}$). CG-Cd: common garden comparing control and Cd-treated plants; CG-Cu: common garden comparing control and Cu-treated plants; Df: degrees of freedom; adj. R^2^: adjusted R^2^.

**Table S5**: Information about the differentially methylated positions (DMPs) between control and Cd-treated, and control and Cu-treated plants from four populations (Sc1 to Sc4) of the copper moss *Scopelophila cataractae*. A summary of the number of DMPs per population, treatment and sequence context (CG, CHG, or CHH) is also presented. contig: contig in which the DMP is located; pos: position of the DMP within the contig; context: sequence context; Sc1/2/3/4_Cd/Cu: methylation level of each cytosine position in the samples treated with Cd or Cu from each population; Sc1/2/3/4_C: methylation level of each cytosine position in the control samples from each population; meth_diff: difference in methylation level between treated and control plants; annotation description: description of the locus to which the contig mapped during the BLAST search; number GOs: number of gene ontology terms resulting from the annotation; GO Ids; gene ontology identification code; GO names: names of the gene ontology terms resulting from the annotation. Underlined DMPs represent DMPs in common to Cd and Cu treatments within each population. **Excel File**.

**Table S6**: Information about all differentially expressed transcripts (DETs) in response to Cu in populations Sc3 and Sc4 of the copper moss *Scopelophila cataractae*. up- and down-regulated transcripts are presented for each population separately, and the DETs in common between the two populations are also presented. contig_ID: ID of the transcript generated during the *de novo* transcriptome assembly; contig_Length: length of the transcript; Description: description of the locus to which the contig mapped during the BLAST search; number GOs: number of gene ontology terms resulting from the annotation; GO Ids; gene ontology identification code; GO names: names of the gene ontology terms resulting from the annotation; Enzyme_Codes: Enzyme Commission (EC) annotation indicating the classification of the transcript based on the overall reaction catalyzed (only available for transcripts with GO annotation); Enzyme_Names: names of the enzymes annotated following the Enzyme Commission (EC) annotation; logFC: log2-transformed fold change between control and treated plants; unshrunk.logFC: unshrunk log2-fold-change; logCPM: log-transformed transcript counts per million; PValue: significance of the test; FDR: false discovery rate. **Excel File**.

**Table S7**: Results of the Fisher’s exact test to look for significantly over- and under-represented gene ontology (GO) terms between the annotated differentially expressed transcripts (DETs) list (test set) and the full list of annotated transcripts (reference set) for each population of the copper moss *Scopelophila cataractae* (Sc3 and Sc4). GO ID: identification code of the over- or under-represented GO term; GO Name: name of the over- or under-represented GO term; GO Category: category to which the GO term belongs including molecular function (MF), biological process (BP), and cellular compartment (CC); P-Value: significance of the test; FDR: false discovery rate. Underlined GOs highlight the under-represented terms in the test set. **Excel File**.

**Table S8**: Functional group classification of down-regulated differentially expressed transcripts (DETs) between Cu-treated and control plants of *Scopelophila cataractae* from this study. **Excel File**.

**Supplementary Figures**

**Figure S1**: Visualization of the genetic structure of four populations of *Scopelophila cataractae* built with the scaled allele frequencies (obtained with the function *scaleGen* from the adegenet R package) from SNPs with no missing values (n=23,252 SNPs).

**Figure S2**: Density plots of the DNA methylation level for each sequence context separately (CG, CHG, CHH) in 30 samples of the copper moss *Scopelophila cataractae* obtained with (A) the complete single methylation polymorphism (SMP) matrix (N=43,689 SMPs), and (B) the complete and polymorphic SMP matrix (N=3,801 SMPs).

**Figure S3**: Average DNA methylation per group (i.e. each unique population and treatment combination) in each separate sequence context (CG, CHG, CHH) and across all contexts (all), and genomic feature (gene, repeat, transposon) in samples of the copper moss *Scopelophila cataractae*. Average methylation across groups is presented within parentheses.

**Figure S4**: Percentage of differentially methylated positions (out of 3,801 polymorphic cytosines) between Cd-treated and control plants, and Cu-treated and control plants for each population of the copper moss *Scopelophila cataractae* (Cd/Cu_vs_C_Sc1/Sc2/Sc3/Sc4). Numbers inside the bars represent the percentage value.

**Figure S5**: Venn diagrams showing the number of unique and shared differentially methylated positions (DMPs) in four populations of the copper moss *Scopelophila cataractae* (Sc1 to Sc4) between Cd-treated and control (A) and Cu-treated and control plants (B). The number of unique and shared DMPs between treatments for each population are also shown (C).

**Figure S6**: Expression change (log_2_FC) of differentially expressed transcripts in response to Cu (n=220) shared between populations Sc3 and Sc4 of the copper moss *Scopelophila cataractae*.

**Figure S7**: Number of up- and down-regulated transcripts associated to level 3 gene ontology (GO) terms related to biological process, molecular function, and cellular component in two populations of the copper moss *Scopelophila cataractae* (Sc3 and Sc4). Red GOs: terms commonly up-regulated in the two populations; purple GOs: terms commonly down-regulated in the two populations; black GOs: population-specific up- and down-regulated GO terms; underlined GOs: terms that were both up- and down-regulated transcripts within each population.

Table S1: Total number of sequencing reads (in million) per sample, group and population of the copper moss *Scopelophila cataractae* in the epiGBS dataset after demultiplexing. Bold sample IDs highlight samples discarded during the second step of the filtering (see section 2.5.1 of the manuscript). C: control samples; Cd: cadmium-treated samples; Cu: copper-treated samples.

| Sample ID | Population | Treatment | Per sample | Per group | Per population |
| --- | --- | --- | --- | --- | --- |
| Sc1_C1 | Sc1 | C | 4.13 | 7.79 | 20.37 |
| Sc1_C2 |  |  | 2.64 |  |  |
| **Sc1_C3** |  |  | 1.02 |  |  |
| **Sc1_Cd1** |  | Cd | 1.14 | 6.39 |  |
| Sc1_Cd2 |  |  | 1.38 |  |  |
| Sc1_Cd3 |  |  | 3.87 |  |  |
| Sc1_Cu1 |  | Cu | 2.55 | 6.19 |  |
| Sc1_Cu2 |  |  | 1.85 |  |  |
| Sc1_Cu3 |  |  | 1.80 |  |  |
| **Sc2_C1** | Sc2 | C | 0.60 | 4.68 | 20.83 |
| Sc2_C2 |  |  | 2.26 |  |  |
| Sc2_C3 |  |  | 1.82 |  |  |
| Sc2_Cd1 |  | Cd | 3.23 | 7.97 |  |
| Sc2_Cd2 |  |  | 2.29 |  |  |
| Sc2_Cd3 |  |  | 2.45 |  |  |
| Sc2_Cu1 |  | Cu | 2.16 | 8.18 |  |
| Sc2_Cu2 |  |  | 2.22 |  |  |
| Sc2_Cu3 |  |  | 3.79 |  |  |
| Sc3_C2 | Sc3 | C | 2.32 | 4.03 | 20.58 |
| Sc3_C3 |  |  | 1.71 |  |  |
| Sc3_Cd1 |  | Cd | 2.57 | 6.93 |  |
| **Sc3_C2** |  |  | 0.96 |  |  |
| Sc3_Cd3 |  |  | 3.41 |  |  |
| Sc3_Cu1 |  | Cu | 3.07 | 9.61 |  |
| Sc3_Cu2 |  |  | 3.69 |  |  |
| Sc3_Cu3 |  |  | 2.85 |  |  |
| Sc4_C1 | Sc4 | C | 1.66 | 4.87 | 23.97 |
| Sc4_C2 |  |  | 1.67 |  |  |
| Sc4_C3 |  |  | 1.55 |  |  |
| Sc4_Cd1 |  | Cd | 4.49 | 11.58 |  |
| Sc4_Cd2 |  |  | 2.73 |  |  |
| Sc4_Cd3 |  |  | 4.36 |  |  |
| Sc4_Cu1 |  | Cu | 2.56 | 7.52 |  |
| **Sc4_C2** |  |  | 1.75 |  |  |
| Sc4_Cu3 |  |  | 3.21 |  |  |

Table S2: Total number of sequencing reads (in million) per sample, population, and group of the copper moss *Scopelophila cataractae* in the RNA-seq dataset before (raw reads) and after (trimmed reads) quality trimming. C: control samples; Cu: copper-treated samples.

| Sample ID | Population | Treatment | Raw | Trimmed | Per group | Per population |
| --- | --- | --- | --- | --- | --- | --- |
| Sc3_C1 | Sc3 | C | 9.7 | 7.9 | 25.0 | 51.0 |
| Sc3_C2 |  |  | 12.2 | 10.2 |  |  |
| Sc3_C3 |  |  | 8.4 | 6.9 |  |  |
| Sc3_Cu1 |  | Cu | 9.9 | 8.2 | 26.0 |  |
| Sc3_Cu2 |  |  | 11.2 | 9.1 |  |  |
| Sc3_Cu3 |  |  | 10.7 | 8.7 |  |  |
| Sc4_C1 | Sc4 | C | 12.2 | 10.0 | 26.5 | 50.8 |
| Sc4_C2 |  |  | 11.5 | 9.4 |  |  |
| Sc4_C3 |  |  | 8.9 | 7.1 |  |  |
| Sc4_Cu1 |  | Cu | 8.3 | 7.0 | 24.3 |  |
| Sc4_Cu2 |  |  | 9.8 | 7.9 |  |  |
| Sc4_Cu3 |  |  | 11.4 | 9.4 |  |  |

Table S3: De novo transcriptome assembly stats from *Scopelophila cataractae* this study **(excel file)**.

Table S4: Results of the distance-based redundancy analysis (dbRDA) carried out to test the effect of population, treatment, and their interaction on genome wide DNA methylation for each of the common garden experiments for each DNA sequence context separately (CG, CHG, CHH), and across all contexts together (all). The results of the permutation test (n = 9999) for the significance of each of the predictors are also shown (Predictors). This analysis was run on two different response variables: the pairwise distance matrix calculated with the methylation level only ($d_{st}^{m}$), and the pairwise distance matrix calculated with the methylation level and variance ($d_{st}^{m\&v}$). CG-Cd: common garden comparing control and Cd-treated plants; CG-Cu: common garden comparing control and Cu-treated plants; Df: degrees of freedom; adj. R^2^: adjusted R^2^.

| Response | Experiment | Context | Predictor | Df | Variance | F | Pr(>F) | adj. R2 |
| --- | --- | --- | --- | --- | --- | --- | --- | --- |
| $d_{st}^{m}$ | CG-Cd | all | model | 7 | 13.32 | 1.03 | 0.229 | - |
|  |  |  | residual | 11 | 20.42 |  |  |  |
|  |  | CG | model | 7 | 13.46 | 1.02 | 0.347 | - |
|  |  |  | residual | 11 | 20.81 |  |  |  |
|  |  | CHG | model | 7 | 13.08 | 1.02 | 0.342 | - |
|  |  |  | residual | 11 | 20.23 |  |  |  |
|  |  | CHH | model | 7 | 13.38 | 1.03 | 0.207 | - |
|  |  |  | residual | 11 | 20.36 |  |  |  |
|  | CG-Cu | all | model | 7 | 12.56 | 0.99 | 0.528 | - |
|  |  |  | residual | 12 | 21.65 |  |  |  |
|  |  | CG | model | 7 | 12.92 | 1.01 | 0.440 | - |
|  |  |  | residual | 12 | 22.02 |  |  |  |
|  |  | CHG | model | 7 | 11.62 | 1.01 | 0.371 | - |
|  |  |  | residual | 12 | 19.68 |  |  |  |
|  |  | CHH | model | 7 | 12.83 | 0.98 | 0.610 | - |
|  |  |  | residual | 12 | 22.36 |  |  |  |
| $d_{st}^{m\&v}$ | CG-Cd | all | model | 7 | 11405 | 5.08 | 1.0E-04 | 61.3 |
|  |  |  | residual | 11 | 3531 |  |  |  |
|  |  | CG | model | 7 | 10952 | 4.89 | 1.0E-04 | 60.2 |
|  |  |  | residual | 11 | 3520 |  |  |  |
|  |  | CHG | model | 7 | 11006 | 4.76 | 1.0E-04 | 59.4 |
|  |  |  | residual | 11 | 3630 |  |  |  |
|  |  | CHH | model | 7 | 11775 | 5.31 | 1.0E-04 | 62.6 |
|  |  |  | residual | 11 | 3487 |  |  |  |
|  | CG-Cu | all | model | 7 | 10859 | 4.48 | 1.0E-04 | 56.2 |
|  |  |  | residual | 12 | 4152 |  |  |  |
|  |  | CG | model | 7 | 10233 | 4.82 | 1.0E-04 | 58.5 |
|  |  |  | residual | 12 | 3641 |  |  |  |
|  |  | CHG | model | 7 | 9439 | 4.61 | 1.0E-04 | 57.1 |
|  |  |  | residual | 12 | 3509 |  |  |  |
|  |  | CHH | model | 7 | 11795 | 4.34 | 1.0E-04 | 55.1 |
|  |  |  | residual | 12 | 4663 |  |  |  |

Table S5: Complete set of differentially methylated cytosine positions (DMPs) between control and treated plants of *Scopelophila cataractae* from this study **(excel file)**.

Table S6: Complete set of differentially expressed transcripts (DETs) between control and treated plants of *Scopelophila cataractae* from this study. DETs between control plants of the two populations are also shown **(excel file)**.

Table S7: Complete results of the Fisher exact test for over- and under-represented gene ontology (GO) terms between the annotated differentially expressed transcripts (DETs) list and the full list of annotated transcripts between treated and control plants of *Scopelophila cataractae* from this study. Represent under-represented GO terms are underlined **(excel file)**.

Table S8: Functional group classification of down-regulated differentially expressed transcripts (DETs) between Cu-treated and control plants of *Scopelophila cataractae* from this study **(excel file)**.

**Figures**

Figure S1: Visualization of the genetic structure of four populations of *Scopelophila cataractae* built with the scaled allele frequencies (obtained with the function *scaleGen* from the adegenet R package) from SNPs with no missing values (n=23,252 SNPs).

Figure S2: Density plots of the DNA methylation level for each sequence context separately (CG, CHG, CHH) in 30 samples of the copper moss *Scopelophila cataractae* obtained with (A) the complete single methylation polymorphism (SMP) matrix (n=43,365 SMPs), and (B) the complete and polymorphic SMP matrix (n=3,769 SMPs).

Figure S3: Average DNA methylation per group (i.e. each unique population and treatment combination) in each separate sequence context (CG, CHG, CHH) and across all contexts (all), and genomic feature (gene, repeat, transposon) in samples of the copper moss *Scopelophila cataractae*. Average methylation across groups is presented within parentheses.

Figure S4: Percentage of differentially methylated positions (out of 3,769 polymorphic cytosines) between Cd-treated and control plants, and Cu-treated and control plants for each population of the copper moss *Scopelophila cataractae* (Cd/Cu_vs_C_Sc1/Sc2/Sc3/Sc4). Numbers inside the bars represent the percentage value.

Figure S5: Venn diagrams showing the number of unique and shared differentially methylated positions (DMPs) in four populations of the copper moss *Scopelophila cataractae* (Sc1 to Sc4) between Cd-treated and control (A) and Cu-treated and control plants (B). The number of unique and shared DMPs between treatments for each population are also shown (C).

Figure S6: Expression change (log_2_FC) of differentially expressed transcripts in response to Cu (n=220) shared between populations Sc3 (red line) and Sc4 (green line) of the copper moss *Scopelophila cataractae*.

Figure S7: Number of up- and down-regulated transcripts associated to level 3 gene ontology (GO) terms related to biological process, molecular function, and cellular component in two populations of the copper moss *Scopelophila cataractae* (Sc3 and Sc4). Red GOs: terms commonly up-regulated in the two populations; purple GOs: terms commonly down-regulated in the two populations; black GOs: population-specific up- and down-regulated GO terms; underlined GOs: terms that were both up- and down-regulated transcripts within each population.
